# The SCIentinel study - prospective multicenter study to define the spinal cord injury-induced immune depression syndrome (SCI-IDS) - study protocol and interim feasibility data

**DOI:** 10.1186/1471-2377-13-168

**Published:** 2013-11-09

**Authors:** Marcel A Kopp, Claudia Druschel, Christian Meisel, Thomas Liebscher, Erik Prilipp, Ralf Watzlawick, Paolo Cinelli, Andreas Niedeggen, Klaus-Dieter Schaser, Guido A Wanner, Armin Curt, Gertraut Lindemann, Natalia Nugaeva, Michael G Fehlings, Peter Vajkoczy, Mario Cabraja, Julius Dengler, Wolfgang Ertel, Axel Ekkernkamp, Peter Martus, Hans-Dieter Volk, Nadine Unterwalder, Uwe Kölsch, Benedikt Brommer, Rick C Hellmann, Ramin R Ossami Saidy, Ines Laginha, Harald Prüss, Vieri Failli, Ulrich Dirnagl, Jan M Schwab

**Affiliations:** 1 Department of Neurology and Experimental Neurology, Charité - Universitätsmedizin Berlin, Charitéplatz 1, 10117 Berlin, Germany; 2 Clinical and Experimental Spinal Cord Injury Research (Neuroparaplegiology), Charité - Universitätsmedizin Berlin, Charitéplatz 1, 10117 Berlin, Germany; 3 Department of Musculoskeletal Surgery, Charité - Universitätsmedizin Berlin, Augustenburger Platz 1, 13353 Berlin, Germany; 4 Institute of Medical Immunology, Charité - Universitätsmedizin Berlin, Augustenburger Platz 1, 13353 Berlin, Germany; 5 Berlin-Brandenburg Center for Regenerative Therapies (BCRT), Charité - Universitätsmedizin Berlin, Augustenburger Platz 1, 13353 Berlin, Germany; 6 Department of Immunology, Labor Berlin – Charité Vivantes GmbH, Sylter Straße 2, 13353 Berlin, Germany; 7 Treatment Centre for Spinal Cord Injuries, Trauma Hospital Berlin, Warener Straße 7, 12683 Berlin, Germany; 8 Division of Trauma Surgery, University Hospital of Zürich, Sternwartstrasse 14, 8091 Zurich, Switzerland; 9 Spinal Cord Injury Center, University Hospital Balgrist, Forchstrasse 340, 8008 Zurich, Switzerland; 10 Department of Neurosurgery, University of Toronto, 399 Bathurst St, Toronto, ON M5T 2S8 Canada; 11 Department of Neurosurgery, Charité - Universitätsmedizin Berlin, Augustenburger Platz 1, 13353 Berlin, Germany; 12 Centre for Trauma- and Reconstructive Surgery, Charité - Universitätsmedizin Berlin, Hindenburgdamm 30, 12200 Berlin, Germany; 13 Trauma Surgery and Orthopedics Clinic, Trauma Hospital Berlin, Warener Straße 7, 12683 Berlin, Germany; 14 Department of Clinical Epidemiology and Applied Biostatistics, Eberhard Karls Universität Tübingen, Silcherstraße 5, 72076 Tübingen, Germany; 15 German Center for Neurodegenerative Diseases (DZNE), c/o Charité - Universitätsmedizin Berlin, Charitéplatz 1, 10117 Berlin, Germany; 16 Center for Stroke Research Berlin, Charité - Universitätsmedizin Berlin, Charitéplatz 1, 10117 Berlin, Germany

**Keywords:** Spinal cord injury, Immune paralysis, Lesion height dependency, High-dose methylprednisolone treatment, Infections

## Abstract

**Background:**

Infections are the leading cause of death in the acute phase following spinal cord injury and qualify as independent risk factor for poor neurological outcome (“disease modifying factor”). The enhanced susceptibility for infections is not stringently explained by the increased risk of aspiration in tetraplegic patients, neurogenic bladder dysfunction, or by high-dose methylprednisolone treatment. Experimental and clinical pilot data suggest that spinal cord injury disrupts the balanced interplay between the central nervous system and the immune system. The primary hypothesis is that the **S**pinal **C**ord **I**njury-induced **I**mmune **D**epression **S**yndrome (SCI-IDS) is 'neurogenic’ including deactivation of adaptive and innate immunity with decreased HLA-DR expression on monocytes as a key surrogate parameter. Secondary hypotheses are that the Immune Depression Syndrome is i) injury level- and ii) severity-dependent, iii) triggers transient lymphopenia, and iv) causes qualitative functional leukocyte deficits, which may endure the post-acute phase after spinal cord injury.

**Methods/Design:**

SCIentinel is a prospective, international, multicenter study aiming to recruit about 118 patients with acute spinal cord injury or control patients with acute vertebral fracture without neurological deficits scheduled for spinal surgery. The assessment points are: i) <31 hours, ii) 31–55 hours, iii) 7 days, iv) 14 days, and v) 10 weeks post-trauma. Assessment includes infections, concomitant injury, medication and neurological classification using **A**merican Spinal Injury Association **i**mpairment **s**cale (AIS) and neurological level. Laboratory analyses comprise haematological profiling, immunophenotyping, including HLA-DR expression on monocytes, cytokines and gene expression of immune modulators. We provide an administrative interim analysis of the recruitment schedule of the trial.

**Discussion:**

The objectives are to characterize the dysfunction of the innate and adaptive immune system after spinal cord injury and to explore its proposed 'neurogenic’ origin by analyzing its correlation with lesion height and severity. The trial protocol considers difficulties of enrolment in an acute setting, and loss to follow up. The administrative interim analysis confirmed the feasibility of the protocol. Better understanding of the SCI-IDS is crucial to reduce co-morbidities and thereby to attenuate the impact of disease modifying factors to protect neurological “outcome at risk”. This putatively results in improved spinal cord injury medical care.

**Trial registration:**

DRKS-ID: DRKS00000122 (German Clinical Trials Registry)

## Background

The effective treatment of worldwide 2.5 million paralyzed, spinal cord injured patients represents an unmet medical need to date [1]. In addition, the numbers of non-traumatic cases (e.g. tumor, cervical spondylotic myelopathy) of spinal cord injury (SCI) are increasing. Infections, i.e. pneumonia and urinary tract infections are a leading cause of morbidity and mortality in patients with acute SCI [2,3]. However, attributing infections to motor-paralysis related dysfunction alone does not sufficiently explain the increased susceptibility to develop infections after SCI. Among others, dysphagia occurs also in healthy patients over night without causing pneumonia. Thus, higher rates of dysphagia in SCI patients with cervical lesions do not solely explain increased rates of pneumonia.

It has been elucidated recently that SCI might increase predisposition to infections by Central Nervous System (CNS)-specific mechanisms: CNS-injury induces a disturbance of the normally well-balanced interaction between the immune system and the CNS resulting in a Spinal Cord Injury-induced Immune Depression Syndrome (SCI-IDS) [4-9]. Presence of SCI-IDS has been verified independently after experimental [10] and human SCI [11]. In brief, SCI 'neurogenically’ ablates the immune system and enhances susceptibility to develop infections, which in turn might cause a generalized wound healing impairment – also affecting wound-healing/repair of the spinal cord lesion itself. Of note, besides increasing the mortality, infections represent an independent risk factor for impaired functional neurological recovery e.g. by i) reducing the conversion rate from being 'completely’ to 'incompletely’ paralyzed and ii) impairing gain of American Spinal Injury Association (ASIA) motor scores [12].

Based on preclinical and first clinical observations within a pilot trial [5,6] we aim to develop prognostic surrogate parameters in order to predict and selectively identify patients 'at risk’ to develop infections. Here, we propose to establish parameters after human SCI, which have been tested in other clinical paradigms of elevated infectious risk such as status post cardiopulmonary bypass [13] and after ischemic CNS injury [14,15]. Therefore, we apply methods established in humans, such as human leukocyte antigen (HLA)-DR expression on monocytes [14] and Concanavalin A (ConA)-induced cytokine expression in whole blood samples, as surrogate markers of SCI-IDS. HLA-DR expression on monocytes serves as primary outcome measure. Moreover, a peripheral blood leukocyte mRNA expression profile encoding for immune modulatory proteins will be investigated to sense and therefore predict an evolving immune suppression as early as possible. Furthermore, we will measure the presence of individual predisposition for infections by assessing polymorphisms in coding areas of MHC-proteins, toll-like pattern-recognition receptors and selected cytokines [16].

The prevention of infections facilitated by the 'neurogenic’ immune suppression syndrome (with its pronounced penetrance referred to as 'immune paralysis’) aims to (i) decrease mortality, (ii) reduce expensive hospitalization time and, (iii) improve the functional outcome, since SCI patients suffering infections are prone to develop inferior neurological recovery [12]. Molecular treatment strategies aiming to foster axonal plasticity and repair might be complemented by approaches that intend to protect the intrinsic neurological recovery potential. Given a realistic therapeutic timeframe of opportunity after SCI, the early identification of SCI-patients 'at risk’ would offer a 'preventive’ option to treat infections earlier and thereby avoid their detrimental consequences. This protective strategy might save neurological function 'at risk’ and thereby improve quality of life. In addition, it would be possible to unmask putative confounding factors that influence neurological outcome measures of relevance for interventional or rehabilitative trials [12].

A solid body of evidence demonstrates that SCI is associated with an early onset of immune suppression (SCI-IDS). Identification of SCI patients as immune compromised is a clinically relevant finding, yet widely underappreciated and warranting further analysis. In order to decipher the underlying mechanisms of SCI-IDS we are following-up on experimental results [5,7,10,17], clinical pilot trials [6,8], and cohort studies [9,18,19] with the international SCIentinel trial. We outline the study protocol and design, define the main outcome parameters, and provide the results of an administrative interim analysis.

## Methods/Design

### Study design, study coordination, and participating centers

**Figure 1:**
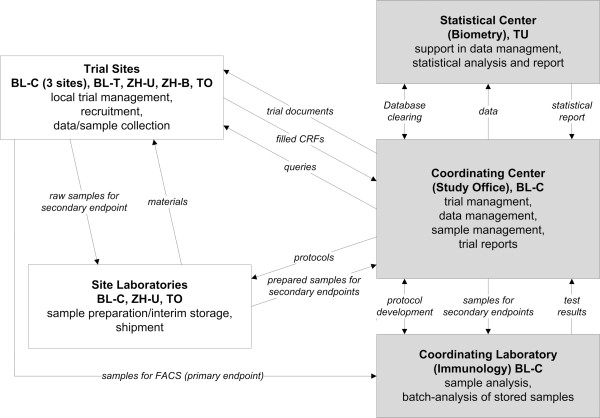
**Multicenter structure and trial management.** Legend: Scheme of the trial workflow. BL-C = Berlin-Charité, BL-T = Berlin-Trauma-Hospital; TU = Biometry Tübingen; ZH-U = Zurich-University Hospital; ZH-B = Zurich-Balgrist; TO = University Hospital Toronto.

The “SCIentinel” trial is designed as a prospective multicenter study for the detailed evaluation of the SCI-IDS (Figure 1). The overall coordination is performed by the Department of Experimental Neurology, Clinical and Experimental Spinal Cord Injury Research (Neuroparaplegiology) at the Charité University Hospital, Campus Mitte, Berlin, Germany.

The study comprises seven recruiting trial centers with a specialization in SCI treatment: Treatment Center for Spinal Cord Injuries, Trauma Hospital Berlin, Germany; Center for Musculoskeletal Surgery (Campus Virchow Clinic); Department of Trauma and Reconstructive Surgery (Campus Benjamin Franklin); Department of Neurosurgery (Campus Virchow Clinic and Campus Benjamin Franklin), Charité University Hospital Berlin, Germany; Division of Trauma Surgery, University Hospital Zurich; Spinal Cord Injury Center, University Hospital Balgrist, Zurich, Switzerland, and the Department of Neurosurgery, Toronto Western Hospital, Toronto, Canada. The study investigators are physicians specialized and experienced in the treatment and rehabilitation of patients with SCI.

### Duration

A recruitment period of 30 months is scheduled. Each patient will be followed-up to three months post-trauma. After completion of recruitment and follow-up, a further six months period is planned for the database clearing, the statistical evaluation and the preparation of the trial report. Enrolment has started in August 2011. Expected study completion date is the first quartile of 2014. Publication of the trial report is scheduled for the end of the year 2014.

### Ethics

The protocol received approval by the local Ethics Committees: Ethical Committee of the Charite – Universitätsmedizin Berlin (EA1/001/09), University Health Network Research Ethics Board, Toronto (REB10-0384-AE), Cantonal Ethics Commission, Zurich (KEK-ZH-Nr. 2011–0059). Participants will be informed about the trial, orally and in written form, using patient’s information sheets and written informed consent will be obtained. This study complies with the Helsinki Declaration, the principles of Good Clinical Practice (GCP) and the Personal Data Protection Act. The study will also be carried out in keeping with local legal and regulatory requirements. The study has been registered in the German Clinical Trials Registry (DRKS-ID: DRKS00000122).

### Participants

**Figure 2:**
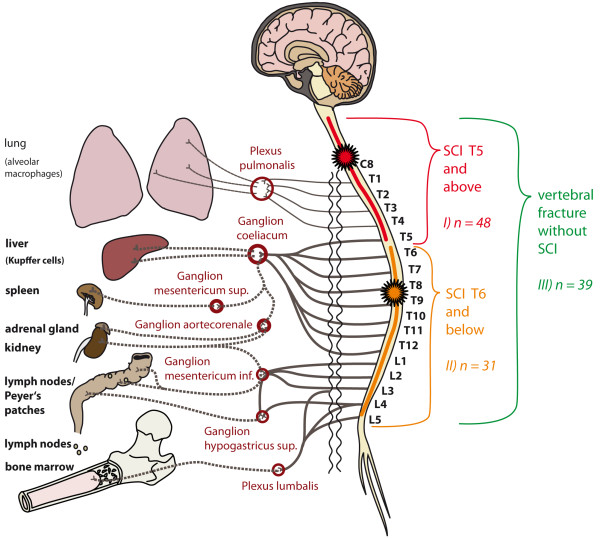
**Relay-interaction of the immune system and the central nervous system.** Legend: Groups for primary comparison in relation to the major sympathetic outflow (vegetative innervation). In Group I (SCI of the neurological level T4 or above) the lesion is localized within the spinal segments C2-T5, resulting in a disturbance of the sympathetic innervation of immunologically relevant organs through the coeliac ganglion and further ganglia connected through lower segments of the spinal cord. Of note, the neurological level is defined by the ISNCSCI as the most cranial segment with normal sensory function and a muscle grade of at least 3/5 with normal function in the segments above on both sides of the body, i.e. in case of a neurological level T4, the lesion begins in the segment T5. In Group II (SCI of the neurological level T5 or below) the lesion is located in the spinal segment T6 or below. Thus, the sympathetic outflow to the coeliac ganglion is expected being only partially disrupted or completely preserved. Group III consists of patients with vertebral fracture alone without injury to the spinal cord (control). Here, the sympathetic innervation is intact.

A number of 118 patients is planned to be allocated to the study for examination of the primary endpoint. In order to allow for investigation of the role of disturbed innervation of the major splanchnic nerve (T5-T9) as linked to alterations of the immune system function, we categorize SCI patients into lesions with the neurological level T4 or above versus lesions neurological level T5 or below. Therefore, the patients will be assigned to 3 groups: i) 48 SCI-patients with a neurological level T4 or above; ii) 31 patients with a neurological level T5 or below; iii) 39 patients with an acute vertebral fracture, but without SCI as control group (Figure 2).

The study will not interfere with methylprednisolone treatment per site regimens and patients will be also enrolled, but are excluded from the analysis of the primary endpoint. Based on experiences from the clinical practice in the recruiting trial centers the rate of cases treated with methylprednisolone is estimated to be 5-10%. Thus, up to 12 patients will additionally be included for explorative examination of methylprednisolone-related alterations of the immune system after SCI (Figure 2).

### Sample size calculation

The primary endpoint is a difference in HLA-DR expression on monocytes exactly quantified by analysing antibody-binding molecules/cell within the first week (day 3–4) post-trauma. Numbers of patients to be enrolled were calculated on the basis of a pilot study (n = 26) [6]. The sample size calculation was performed for an effect size of 0.18. The type one error was be set to 0.05 (two-sided); type two error was set to 0.2. The number of patients eligible for evaluation (for testing the hypothesis by the one way anova) is 56 in total (software nquery, version 7.0). Due to an expected rate of drop-outs/missing data of 50% and non-parametric testing, where applicable, the number of patients to be recruited increases to 118 in total. The expected rate of drop-outs/missing data relates to the structural conditions of acute SCI care in terms of i) delayed transfer to specialized SCI trial centers after primary care in external non-specialized centers and ii) loss to follow up due to discharge from inpatient rehabilitation, particularly in the control group or in cases of mild incomplete SCI. Consequently, to allow for a completion of the study within a feasible time period consideration of missing values is unavoidable.

The sample size calculation was performed taking the asymmetric natural distribution of the neurological lesion level into account [20]. Injury to the cervical and high thoracic spinal cord has the highest incidence and represents the largest group of SCI patients. Thus, the relation of the 3 groups to each other was determined as follows based on the pilot trial data i) 41% (n = 48); ii) 26% (n = 31); iii) 33% (n = 39).

### Enrolment and eligibility criteria

With the seven study centers treating 20 to 60 acute SCI patients admitted to the emergency ward of each center per year we estimate to screen about 200 SCI patients per year and about 40 of them are expected to meet eligibility criteria (Table 1).

**Table 1:** Eligibility criteria

**Inclusion criteria**	
(1)	Patients with acute isolated spinal cord injury (AIS A-D) planned for surgical stabilization and decompression, lesion may include more than 1 segment
(2)	Patients with acute isolated spinal fracture planned for surgical stabilization, lesion may include more than 1 segment
(3)	≥ 2 spinal cord or vertebral lesions definable one from another
(4)	Legal age of the patient
(5)	Documented informed consent of the patient
**Exclusion criteria**	
(1)	Non-traumatic spinal cord injury
(2)	2 or more spinal cord or vertebral lesions definable one from another
(3)	Severe polytrauma *(definition: patients with severe injuries of life-sustaining organ systems, which per se and in the acute phase are life-threatening (e.g., severe pelvic trauma, severe body cavity injuries)*
(4)	Concomitant traumatic brain injury (TBI) *(definition: i) Patients with persisting neurological deficit in consequence of the TBI, ii) patient with severe TBI (Glasgow Coma Scale ≤ 8), and iii) patients with intracranial pressure monitoring sensors.)*
(5)	Neoplasia and/or antineoplastic therapy
(6)	Rheumatic disease, collagenosis, vasculitis or other autoimmune disease
(7)	Preexisting chronic infectious disease (before the injury)
(8)	Preexisting systemic steroid treatment
(9)	Severe alcohol or drug addiction
(10)	Pregnancy, lactation

Investigators will evaluate the patients after admission to the emergency ward for eligibility. Before final inclusion into the study the investigator will conduct an interview with the patients to verify the inclusion- and exclusion-criteria and obtain written informed consent.

### Assessment time points

**Figure 3:**
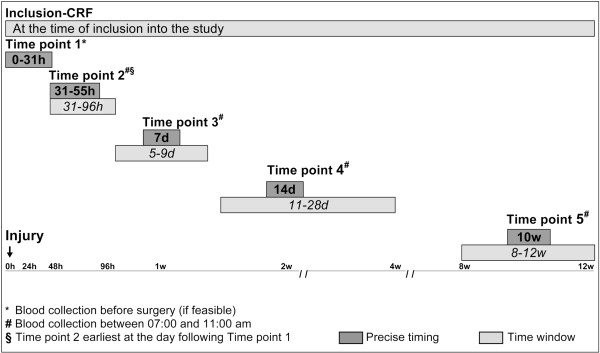
**Longitudinal trial design.** Legend: Assessment points and time frames. The starting point of the time line is the time of injury not the time of surgery. In the case of difficulties in scheduling the visits 2 to 5 may be conducted within the indicated time windows (*italic*). The circadian rhythmics are respected.

Measurements and observations are scheduled in all groups in the same manner (Figure 3). An inclusion assessment will be performed at the study entry and is documented in a case report form (CRF). The baseline CRF includes the neurological classification, assessment of injury date and time, medical history, concomitant injury, acute SCI therapy concerning high-dose methylprednisolone treatment as well as surgical intervention.

At the 5 study visits, equal blood samples and clinical data will be obtained. The clinical data comprise infections, medication, surgery, and blood transfusions. The first assessment should be completed as early as feasible after the injury, preferably before surgery, otherwise at least within 31 hours post-injury. The second assessment point refers to a time window of 31–55 hours post-injury. To ensure a sufficient time interval to the first assessment the second one should be performed on the following day at the earliest. This allows a better intra-individual analysis. The visits 3, 4 and 5 refer to day 7, 14 and to 10 weeks after injury, respectively. Blood withdrawals, except at time point 1 should be performed between 7:00 and 11:00 am. This setting determines the influence of the circadian rhythm to a minimum.

In the case, if it is unavoidable that a patient may be assigned to the study later as 31 hours (delayed recruitment) e.g., due to later admission or transfer to a study center (see also sample size calculation), study inclusion is tolerated at any later time point. In individual cases of delayed recruitment or patient’s absence, documentation of visits 2–5 may be scheduled in extended time windows (Figure 3).

### Definition of infections

The most prevalent infections after SCI, in detail chest infections and urinary tract infections, are diagnosed and documented in the study according to established definitions of disease to ensure a biometric comparability [15,21,22] (Table 2). Additionally, a distinction is made between non-symptomatic and symptomatic urinary tract infections, i.e. the latter are classified with regard to evidence of fever, increased spasticity, bladder spasm, suprapubic or flank pain, autonomic hyperreflexia, malaise, or lethargy. All other infections are documented according to the usual diagnostic criteria of the participating centers.

**Table 2:** Diagnostic criteria for chest infection and urinary tract infection

**Type of infection**	**Criteria**
Chest infection, if ≥ 3 criteria apply	Temperature < 36.0°C or ≥ 37.5°C
	Putrid secretion
	Pathological respiration (rales, bronchial breathing, tachypnea > 22/min)
	Opacities in chest x ray (required for the diagnosis of pneumonia)
	Detection of pathogenic germs in sputum
	pO2 < 70 mmHg/O2 saturation < 93%
Urinary tract infection, if ≥ 1 criteria apply	Bacteriuria with bacterial count > 10^5^ cells/ml
	Leukocyturia ≥ 100 WBC/mm^3^, respectively 100.000 WBC/ml
Symptomatic urinary tract infection, if ≥ 1 criteria apply	≥ 1 criteria for urinary tract infection
	≥ 1 of the following criteria: fever, suprapubic or flank discomfort, bladder spasm, increased spasticity, worsening dysreflexia

### Neurological classification

The neurological evaluation is performed according to the International Standards for Neurological Classification of Spinal Cord Injury (ISNCSCI), a revision of ASIA classification [23,24]. Within the ISNCSCI-regime the ASIA impairment scale (AIS) as a measure for completeness of the injury, and the single neurological level of the lesion are assessed.

### Blood sample handling

**Figure 4:**
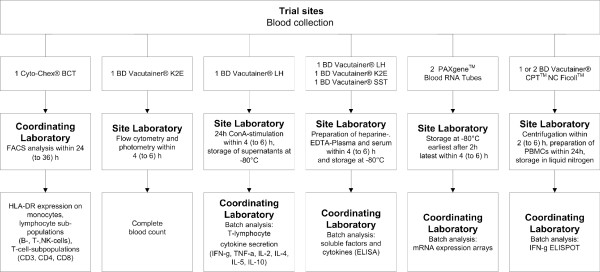
**SCI-IDS Immunephenotyping – qualitative and quantitative measures.** Legend: Scheme of blood collection, sample preparation and analysis procedures. ConA = Concanavalin A, ELISA = Enzyme Linked Immunosorbent Assay, ELISPOT = Enzyme Linked Immunosorbent Spot, FACS = Fluorescence-activated cell sorting, IFN-g = Interferon-gamma, TNF-g = Tumor necrosis factor – gamma.

Peripheral blood is collected under sterile conditions from each participant at each visit. All samples are labeled with a six-digit pseudonym and any personal information of the participants is removed. Overall, one 2 ml and one 6 ml Ethylenediaminetetraacetic acid (EDTA) BD Vacutainer®, two 3 ml Heparin BD Vacutainer® and one 8.5 ml Serum BD Vacutainer® are needed. Furthermore one 5 ml Cyto-Chex® blood collection tube (BCT) and two 2.5 ml PAXgene™ RNA tubes are collected (Figure 4). Blood samples for flow cytometric analysis are analyzed as soon as possible, preferably within 24 hours latest within 48 hours. Preservation of intracellular and extracellular surface markers is ensured by using Cyto-Chex® BCT [25,26].

Supernatants from whole blood stimulation and further blood samples for secondary outcome measures are stored at -80°C for subsequent batch analysis (Figure 4). In the trial centers at Berlin and Zurich, one additional 8 ml BD Vacutainer® CPT™/Ficoll™ tube is collected for immediate preparation of Peripheral Blood Mononuclear Cells (PBMCs) for storage in liquid nitrogen. The handling of samples follows identical standard operating procedures and checklists at all trial centers.

### Immune profiling of SCI-IDS

The following investigations are performed to determine the immune status: i) immunophenotyping with lymphocyte subpopulations (B, T, NK cells), T cell subpopulation (CD3, CD4, CD8) and monocytic HLA-DR expression; ii) ex vivo T lymphocyte cytokine secretion such as ConA-induced IFN-g, TNF-α, IL-2, IL-4, IL-5, IL-10 secretion and staphylococcal enterotoxin B (SEB)-induced IL-17 production; iii) gene expression analysis for immune activation/deactivation related genes (mRNA expression); iv) circulating immune modulators: such as IL-6, epinephrine; v) indirect infection parameters: procalcitonin, C-reactive protein (CRP), LBP (lipopolysaccharide-binding protein).

Immunophenotyping using fluorescence-activated cell sorting (FACS) including quantitative measurement of monocytic HLA-DR expression as the primary outcome measure will be conducted according to standardized operation procedures established at the Institute of Medical Immunology (Charité - Universitätsmedizin Berlin, Germany) for clinical diagnostics of an immune suppression state [14]. HLA-DR belongs to the MHC class II molecules that are responsible for antigen presentation to CD4+ T-cells. Monocytic HLA-DR expression reflects the state of the adoptive immune competence as it is positively regulated by Th1 cytokines and negatively regulated by anti-inflammatory cytokines, stress hormones and some exogenous agents. Furthermore, monocytic HLA-DR expression is associated with the ability to produce pro-inflammatory mediators upon challenge by bacterial products such as lipopolysaccharides (LPS). Reduced monocytic HLA-DR expression has been established to correlate with an increased risk for infections in critically ill patients previously [14,27,28].

### Quantitative measures of HLA-DR expression

In contrast to the pilot study that measured HLA-DR expression on different cell types [6], the SCIentinel study measures quantitatively the amount of HLA-DR molecules per monocyte (in estimated numbers per cell). The reliability of the method has been previously approved in an inter-laboratory study [14].

The principle of the test is as follows. A mixture of Phycoerythrin (PE) beads conjugated with defined amounts of PE molecules is measured at the same instrument settings as the cells incubated with a mix consisting of anti-human HLA-DR-PE, anti-human CD14-PerCP-Cy5.5, and an inhibitor of HLA-DR turnover (BD Quantibrite™ HLA-DR/Monocyte reagent, BD Biosciences). The PE beads facilitate conversion of the FL2 axis into PE molecules bound per cell. The known ratio of PE to anti-HLA-DR antibody is used to convert the PE molecules per cell into antibodies per cell (AB/c). The anti-HLA-DR antibody, clone L243, reacts with a non-polymorphic HLA-DR epitope and is conjugated with PE molecules in a ratio of 1:1. The anti-CD14 antibody, clone MοP9, is conjugated with PerCP-Cy5.5. CD14 is expressed by the majority of monocytes. In addition, since the cyan dye component of PerCP-Cy5.5 binds to CD64, the anti-CD14 PerCP-Cy5.5 antibody detects all monocytes (CD14 brightly positive and weakly positive). The immunodiagnostical thresholds delineating a graded SCI-IDS penetrance are defined as indicated in Table 3 and were developed based on previous clinical trials after CNS injury [27].

**Table 3:** Thresholds for the interpretation of the analysis of HLA-DR expression on monocytes

**Antibodies/cell**	**Interpretation**
> 15 000	Normal diagnostic findings
10 000 – 15 000	Immunodepression
5 000–10 000	Borderline immunoparalysis
< 5 000	Immunoparalysis

### Data management

Data are collected on a paper CRF (pCRF) basis. All patient’s data are managed with a six-digit pseudonym. At the coordinating center personnel familiar with the trial protocol check the filled pCRFs for completeness and consistency. Implausible or missing data may be corrected or added after consulting the investigator at the trial site through the study management (Queries). The corrected documents will be archived together with the completed CRFs. Data are entered and stored electronically in a database (Access) and are independently double-checked for correctness. The database has secured and restricted access centralized at IT-Center of Charité-Universitätsmedizin Berlin, Germany. Data backups are performed on a daily basis.

### Interim evaluation of recruitment status

An administrative interim evaluation for control of the estimated recruitment schedule after enrolment and follow up of approximately 50% of the calculated sample size has been performed. Patients recruited until April 2013 and followed up to July 2013 were included. The groups for comparison were analyzed for their concordance with the estimated sample size (Table 4). Furthermore the completeness of data for the main laboratory outcome parameters (flow cytometry) at each time point was assessed within the 'per protocol’ population (Table 5).

**Table 4:** Administrative interim analysis of the recruitment status for patients without high-dose methylprednisolone treatment

**Groups**	**Overall n (%)**	**SCI T4 and above n (%)**	**SCI T5 and below n (%)**	**Control group n (%)**	**Drop out n**
**Estimated sample size**	118 (100)	48 (100)	31 (100)	39 (100)	n.a.
**Enrolled partients**	60 (51)	28 (58)	11 (35)	17 (44)	4

**Legend:** Sixty patients without high-dose methylprednisolone treatment were included into the SCIentinel trial from August 2011 to April 2013. The number of patients recruited into the group of SCI patients of the neurological level T5 or below was slightly smaller than expected. This does not match the natural distribution of the lesion level and seems to be random. In total, four patients (7%) dropped out of the interim analysis due to occurrence of exclusion criteria.

**Table 5:** Administrative interim analysis of the completeness of laboratory data (flow cytometry) for each visit relative to the number of enrolled patients feasible for 'per protocol’ analysis

**Groups**		**Visit 1**	**Visit 2**	**Visit 3**	**Visit 4**	**Visit 5**
**Overall** n = 56	Complete data n	32	38	44	44	25
	(% of 'per protocol’ population)	(57)	(68)	(79)	(79)	(45)
**SCI T4 and above** n = 28	Complete data n	17	20	22	22	16
	(% of 'per protocol’ population)	(61)	(71)	(79)	(79)	(57)
**SCI T5 and below** n = 11	Complete data n	6	6	8	10	5
	(% of 'per protocol’ population)	(55)	(55)	(73)	(91)	(45)
**Control group** n = 17	Complete data n	9	12	14	12	4
	(% of 'per protocol’ population)	(53)	(71)	(82)	(71)	(24)

**Legend:** The completeness of the main laboratory data was calculated for the three groups relative to the number of enrolled patients feasible for 'per protocol’ analysis. The sample size calculation takes a 50% rate of drop out or missing values into account. Considering the drop out rate < 10% at the current stage of the trial, a 60% rate of data completeness is acceptable. Critical rates of overall completeness are observed at visit 1 and visit 5 and relate mainly to the control group and to patients with neurological level T5 or below.

### Statistical analysis

Data analysis of the primary endpoint consists of a comparison, between the 3 groups at the early and subsequent follow-up assessment points using one-way ANOVA or nonparametric tests, if appropriate. The analysis will be performed using the full dataset comprising all patients included according to the criteria as defined in the study protocol. In addition, in patients with complete intra-individual datasets also changes over time within the groups will be assessed with descriptive methods. Furthermore, in the case of a relevant amount of missing values, multiple imputation will be taken into consideration for verification of the analysis of the original data. Data obtained from patients treated with methylprednisolone will be primarily analyzed in a casuistic manner.

The statistical analysis of the administrative interim evaluation has been performed using SPSS for Macintosh, Version 19.0.

## Discussion

The SCIentinel trial aims to define SCI-IDS characteristics. We investigate the hypothesis that SCI-IDS is not restricted to quantitative immune suppression (cell depletion) [5-8], but also affects qualitative immune function of 'spared’ immune cells. Qualitative dysfunction of immune cells translates into suppressed innate and adaptive immune cell function that finally leads to impaired host defense [29]. Qualitative effects may extend into chronic SCI [29] and therefore the identification of patients prone to develop infections is also relevant during long-term follow up. Moreover, according to the proposed 'neurogenic’ origin of the SCI-IDS we suggest rostral and complete spinal cord lesions to be associated with a more severe immune depression syndrome compared to more caudal or incomplete lesions. Furthermore we intend to investigate a putative sustained extension into chronic disease phases after SCI. Together, this is embedded the longitudinal prospective trial design and in a power calculation that takes differences between rostral and caudal neurological levels into account. In addition, the impact of facultative co-treatment with high dose methylprednisolone, which may further aggravate SCI-IDS is accessible for descriptive analysis.

### Spine trauma

In order to differentiate the SCI-related 'neurogenic’ effect on the immune system from the 'non-neurogenic’ effect of the surgical intervention/trauma itself (activation of the stress axis/post-aggression syndrome) the study protocol incorporates a control group of patients with isolated vertebral fracture without neurological deficit. Of note, this refinement of the control group definition by the SCIentinel trial overcomes a weakness of the pilot study where patients after smaller surgical interventions such as arthroscopy served as control group [6], which only insufficiently mimics the post-aggression syndrome caused by surgical stabilization of the spine.

In order to evaluate the impact of the lesion height we assess differences between rostral and caudal spinal cord lesions. Consequently, SCI patients were divided into two groups with/without expected impairment of the major sympathetic outflow and compared with the controls applying a three-group strategy for analysis (Figure 1). To assess the effect of lesion severity, we compare complete (AIS A) with incomplete lesions (AIS B-D) in a descriptive sub-group analysis.

### Eligibility criteria

We defined the eligibility criteria (Table 1) in order to minimize inclusion bias and to obtain 'representative’ data. The definitions stipulated in the SCIentinel protocol are consistent with previously published studies [6,8,18,30]. It was necessary to exclude patients with severe, life threatening polytrauma or severe TBI in order to limit variability caused by concomitant injury. For detection of immune system functionality, it is furthermore essential to exclude all circumstances combined with pre-existing alterations in the immunological profile such as chronic autoimmune diseases and pre-existing systemic treatment with corticosteroids.

The administrative interim analysis of the SCIentinel trial has confirmed the feasibility of the eligibility criteria. From August 2011 to April 2013 n = 64 patients have been enrolled for the study which represent 37 patients per year. Thus, the enrolment status is within the expected range. Four of the 64 patients (6%) were treated with high-dose methylprednisolone. Therefore, those patients are to be excluded from the 'per protocol’ analysis. However, casuistic studies on immunosuppressive effects of methylprednisolone treatment in the context of the SCI-IDS are enabled. The remaining 60 patients represent 51% of the estimated sample size (Table 4).

### Observation period

Observational time points (Figure 2) range from acute to sub-acute stages after SCI comparable to the pilot study [6]. Here, the time schedule corresponded to the hallmarks of parenchymal inflammation following SCI. The implementation of sub-acute assessments is in line with previous trials that demonstrated mid- and long-term alterations of the immune system after SCI [8,18,19].

The setting of defined time corridors instead of time points provides a more complete tracking of the participants. In order to prevent a high amount of missing datasets due to loss to follow-up, the protocol allows an inclusion at each documentation time point.

The administrative interim analysis of data completeness for the main laboratory parameters indicates - depending on the group or visit – a completeness of data between 24% and 91% with critical rates of completeness at visit 1 and visit 5, particularly in the control group (Table 5). As a consequence, the recruiting centers have been requested to enroll the patients as soon as possible after injury. Additionally, we intend to address the challenge of loss to follow up by travelling to external rehabilitation centers or by phone calls to remind patients of their study visit at the outpatient clinic. The rate of complete longitudinal datasets ranges between 18% and 36% but is expected to increase during the further trial course since trial routines are better established. Noteworthy, the sample size calculation takes 50% of missing values into account and was performed for one-way ANOVA. Repeated measure analysis requires lower sample sizes. Therefore, it seems feasible that a relevant amount of complete longitudinal datasets can be achieved also for the description of the intra-individual course of the SCI-IDS. Pursuing this study concept, serial investigations on short- and mid-term changes of the immunological function are provided.

### Clinical observations

For the clinical research, it is imperative to use reliable reproducible clinical tests. The ISNCSCI standards revised over the years to provide better, more specific definitions [23,24]. We incorporated the AIS and the single neurological level into the trial protocol for neurological baseline characterization of SCI patients.

The determination of infections is of crucial importance in this study. Prevailing infectious complications of SCI patients are pulmonary and urinary tract infections [4]. Cameron and colleagues recently provided a review consisting of 12 articles of urinary tract infection (UTI) screening for SCI [31]. Noteworthy, there is no universal definition of UTI used in the literature allowing substantial heterogeneity. The American Paraplegia Society (APS) recommends to base the diagnosis of UTI solely on bacteriuria, with a threshold of 10^2^ colony-forming units (cfu)/mL for SCI patients [32]. However, up to 75% of samples taken from asymptomatic SCI male patients under intermittent catheterization contain ≥ 10^2^ cfu/mL [32]. Thus, a threshold of 10^2^ cfu/mL is associated with low specificity, leading to an excess of treatment, with the risk of the emergence of resistant bacteria, adverse events, and unnecessary expense. In consequence, we defined according to the National group on urologic rehabilitation of paraplegics urinary tract infection as bacteriuria greater than 10^5^ cells/ml or WBC count of more than 100/mm^3^[21].

Regarding the development of chest infection criteria defined by Mann et al. were incorporated [22]. For the diagnosis of pneumonia the finding of infiltrates or opacity in chest X-ray will be required consistent with Haeusler et al. who used similar criteria in the analysis of cellular immunodepression preceding infectious complications after acute ischemic stroke [15].

In summary, the clinical assessments and definitions used in the SCIentinel study allow for collection of reliable and reproducible data.

### Primary outcome measure

For meeting the purpose of a more comprehensive characterization of the SCI-IDS and establishing prognostic surrogate parameters, it is essential to implement specific parameters, readily validated in clinical immunology, robust and reproducible [14]. Quantification of immune cell populations in the blood using FACS is a clinically established and precise method that qualifies as primary outcome measure. Our primary measure is the determination of HLA-DR-molecules per monocyte. Alterations in HLA-DR expression on monocytes occur early enough to identify patients at high risk for relevant immune depression and consecutive development of infections. Significantly decreased monocytic HLA-DR levels have been observed as early as day one after acute cerebral ischemia in patients who developed infectious complications in comparison to patients with an uncomplicated clinical course [27]. Furthermore, the method is already established and available in centralized immunological laboratories. It has sufficient diagnostic specificity as a basis for clinical decision-making and therefore is clinically highly relevant. Moreover, we implemented a large setting of secondary endpoints for the development of very early predictive parameters, for example on the level of mRNA expression.

### Possible consequences

The objective of this study is the longitudinal characterization of immunological markers of the innate and acquired immune system after traumatic SCI. Furthermore, the aim is to detect diagnostic surrogate parameters for the clinical application after SCI. The characterization of a causative relationship between the lesion level and/or SCI severity and the type and/or extent of the immune response after SCI - including a critical characterization of the methylprednisolone effect - is essential for the implementation of therapeutic studies. Understanding and recognition of the immunological dysfunction and the altered susceptibility to infections may then assist in consecutive decision-making. This is relevant for the stratification of patients who are at high or low risk for infectious complications [33]. In addition, these findings may also hold the key for early therapeutic immunomodulation aiming to improve overall survival by anticipation and prevention of life-threatening infectious complications.

## Abbreviations

ASIA: American Spinal Injury Association; AIS: ASIA impairment scale; BCT: Blood collection tube; CON A: Concanavalin A; CRF: Case report form; CRP: C-reactive protein; FACS: Fluorescence-activated cell sorting; EDTA: Ethylenediaminetetraacetic acid; GCP: Good clinical practice; HLA: Human leukocyte antigen; ISNCSCI: International standards for neurological classification of spinal cord injury; LPS: Lipopolysaccharides; PBMC: Peripheral blood mononuclear cell; PE: Phycoerythrin; SCI: Spinal cord injury; SCI-IDS: Spinal cord injury – induced immune depression syndrome; SEB: Staphylococcal enterotoxin B; TBI: Traumatic brain injury; UTI: Urinary tract infection.

## Competing interests

The authors declare that they have no competing interests.

## Authors’ contributions

MAK, CD, CM, JMS designed the trial protocol and wrote the first draft of the manuscript. TL, PC, AN, GAW, AC, GL, NN, MGF, PM, HDV, UD reviewed the trial protocol. All authors critically revised the manuscript draft for important intellectual content. MAK, CM, PC, HDV, BB, HP, VF, JMS contributed to the establishment of analytical methods. MAK, CD, TL, EP, RW, PC, AN, KDS, GAW, AC, GL, NN, MGF, PV, MC, JD, WE, AE, NU, UK, RH, RROS, IL, VF JMS substantially contributed to the sample and data acquisition. MAK, CD, HP, VF, JMS were responsible for overall trial coordination and supervision. MAK, CD, RW, PC, GL, NN, MC, BB, RCH, RROS, IL, VF were responsible for local trial management. CM, PC, MGF, PM, HDV, UD provided technical support. MAK, RW were responsible for data management. MK performed the interim feasibility analysis. JMS obtained funding. All authors have read and approved the final version of the manuscript.

## Pre-publication history

The pre-publication history for this paper can be accessed here:

http://www.biomedcentral.com/1471-2377/13/168/prepub
